# An explanatory sequential mixed-methods design to establish thresholds of within-individual meaningful change on a sleep disturbance numerical rating scale score in atopic dermatitis

**DOI:** 10.1007/s11136-022-03294-w

**Published:** 2022-11-22

**Authors:** Carla Dias-Barbosa, Jorge Puelles, Fatoumata Fofana, Sylvie Gabriel, Danielle Rodriguez, Rajeev Chavda, Christophe Piketty

**Affiliations:** 1 Evidera, Ivry-Sur-Seine, France; 2 Galderma, La Tour-de-Peilz, Switzerland; 3 Evidera, Bennekom Born, The Netherlands; 4 Evidera, Seattle, WA USA

**Keywords:** Meaningful within-patient change, Multi-stage mixed methods design, Mixed-methods research, Triangulation, Sleep disturbance, Atopic dermatitis

## Abstract

**Purpose:**

Establishing a meaningful within-individual change (MWIC) threshold is a key aspect for interpreting scores used as endpoints for evaluating treatment benefit. A new patient-reported outcome (PRO), a sleep disturbance numerical rating scale (SD NRS), was developed in adults and adolescents with moderate-to-severe atopic dermatitis (AD). This research aims to establish a MWIC threshold of the SD NRS score in the context of a drug development program.

**Methods:**

An explanatory sequential mixed-methods design was used to address the research objective. This mixed-methods design used phase IIb data and a stand-alone qualitative study. Quantitative anchor-based and distribution-based approaches supported by qualitative-based approaches were conducted, and results were triangulated to determine preliminary MWIC thresholds of the SD NRS score.

**Results:**

Triangulation of results from both quantitative and qualitative approaches suggested that a 2- to 6-point decrease in the SD NRS score change constitutes a preliminary range of MWIC threshold estimates.

**Conclusion:**

This research determined MWIC threshold estimates for the SD NRS score in both adolescents and adults with moderate-to-severe AD using an explanatory sequential mixed-methods design. This mixed-methods design provides interesting insights for establishing MWIC thresholds of a PRO score in the context of a drug development program.

## Introduction

Establishing what constitutes a meaningful change score of clinical outcome assessments (COAs), including patient-reported outcomes (PROs), is essential for interpreting results based on COA endpoints used for evaluating treatment benefit. It includes examination of thresholds for within-individual change as well as between-group differences. Interpretation of meaningful change at the individual level (i.e., meaningful within-individual change [MWIC] or responder definition) corresponds to the amount of change in scores reported by any individual over a predetermined time period that should be interpreted as a benefit to patients [1]. Meaningful change can also be interpreted at the group level to evaluate clinically important differences (CID) [2, 3] between two clinically distinct groups (e.g., experimental treatment and placebo) that have meaningful differences in their level of change in scores. Establishing meaningful change of a COA score is commonly assessed using anchor-based and distribution-based methods [2, 4]. These traditional approaches for interpreting COA scores have both advantages and disadvantages that have been discussed elsewhere [2].

Over the last few years, there has been a growing interest in developing novel methods for establishing thresholds for meaningful change of scores such as the use of semi-structured interviews, vignettes (standard setting), surveys, conjoint analysis, and Delphi panels [2, 5]. This includes mixed-methods research (MMR), combining quantitative methods using data on change scores from clinical trials or observational studies and qualitative methods using data from concept elicitation, cognitive interviewing, or exit interviews [6]. MMR is defined as *“a research in which the investigator collects and analyses data, integrates the findings, and draws inferences using both qualitative and quantitative approaches or methods in a single study or program of inquiry”* [7]. MMR has been well established for more than 50 years in the social behavioral sciences [8–11]. In the field of health outcomes research [12, 13], MMR is now well accepted and commonly used [14, 15]. The US Food and Drug Administration (FDA) [16] has even recommended MMR as a research methodology to collect comprehensive patient community input on the burden of disease and current therapy as well as to identify what is important to patients. Furthermore, the US regulators do recognize that emerging approaches such as MMR may be used to triangulate and interpret COA-based endpoint results.

Our work aims to establish MWIC thresholds of a new PRO, a sleep disturbance numerical rating scale (SD NRS^©^), in adults and adolescents with moderate-to-severe atopic dermatitis (AD), using a mixed method approach in the context of a drug development program. To address our objective, we used an explanatory sequential MMR design using different study samples. The MWIC of the SD NRS had not been established prior to this study and therefore needed to be established from the target population using multiple data sources collected at the patient level (phase II data, and qualitative interviews).

## Methods

### MMR design

**Fig. 1:**
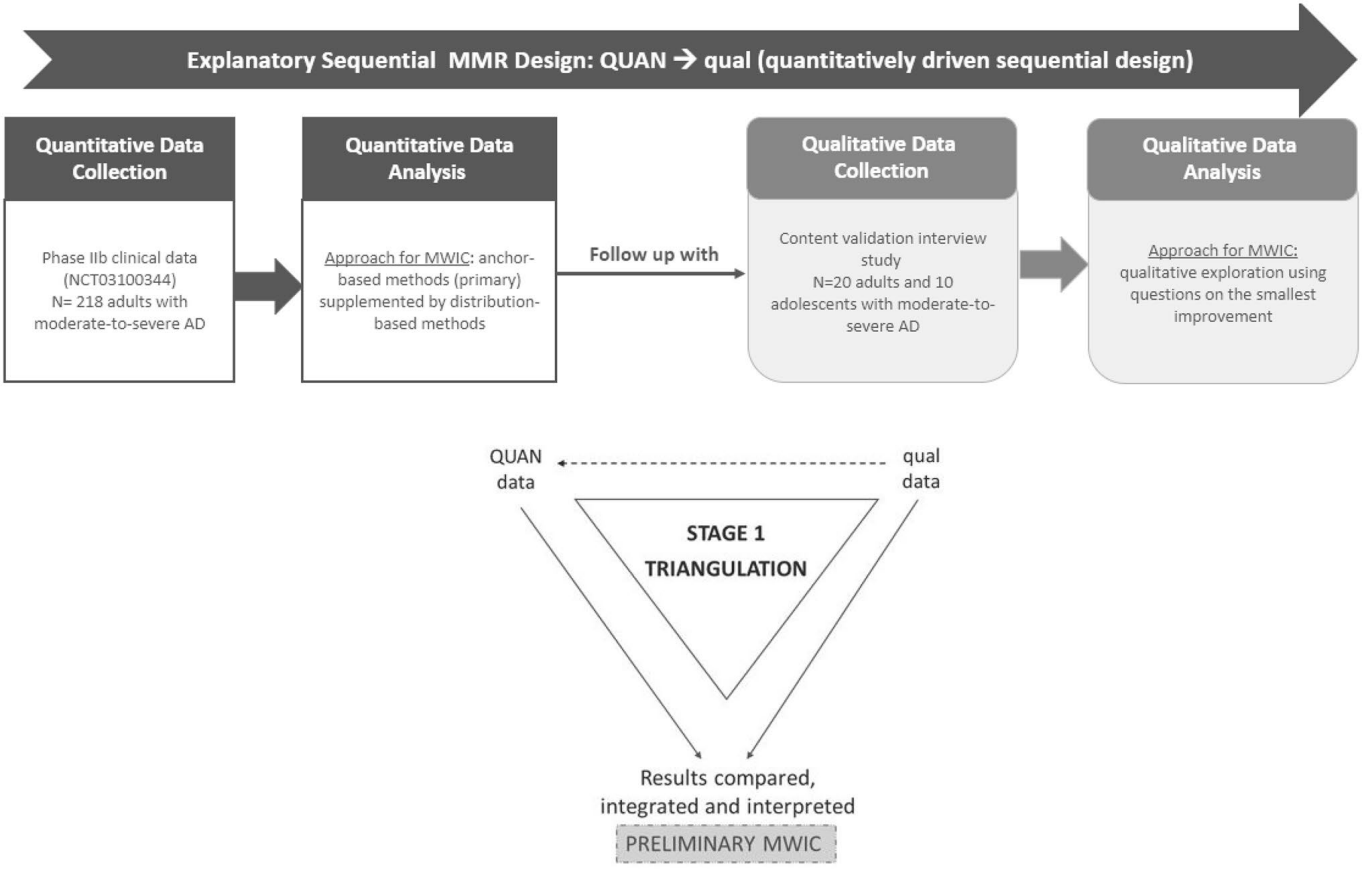
Graph illustration of the explanatory sequential mixed-methods design to establish the MWIC threshold of the SD NRS score. Mixed-methods notation system taken from Morse 1991 [30] and Morse 2003 [31]. The components are indicated as *qual* and *quan* (or QUAL and QUAN to emphasize primacy), respectively, for qualitative and quantitative research. The arrows ( →) refer to sequential implementation of components. *AD* atopic dermatitis; *MMR* mixed-methods research; *MWIC* meaningful within-individual change

Our explanatory sequential MMR design is illustrated in Fig. 1. This design includes two-phases of data collection and analysis conducted sequentially and emphasizes the quantitative approach. It is denoted QUAN→Qual which represents the quantitative study occurs first and has greater weight in addressing our study objective, and the qualitative study follows to explain and support quantitative results. The first phase is a quantitative data collection phase and analysis using SD NRS data collected in a phase IIb clinical trial in moderate-to-severe AD, followed by the second phase of data collection and analysis using qualitative interview data collected with a moderate-to-severe AD population. Results from both phases were analyzed separately and then triangulated to establish preliminary MWIC thresholds on the SD NRS score change (Fig. 1). This research was conducted following the Core Quality Criteria of Mixed Methods Research [17].

### Ethics

The Phase IIb clinical trial involving only adult subjects was conducted in North America (the United States and Canada), Europe (France, Germany, and Poland), and Australia. This clinical study was conducted in accordance with the protocol (RD.03.SPR.114322), the Helsinki declaration (1964) and subsequent amendments, and the International Conference on Harmonization Good Clinical Practice guidelines and in compliance with applicable regulatory requirements. The phase IIb protocol (RD.03.SPR.114322) was approved by the appropriate IECs/IRBs in each country.

The qualitative study protocol involving adult and adolescent subjects in the USA was approved by the Advarra Institutional Review Board in the USA (Columbia, MD). All recruitment procedures complied with current Health Insurance Portability and Accountability Act regulations in the USA. Adult participants had to provide written informed consent prior to study procedures. Adolescent participants had to provide informed assent, and their parent or legal guardian had to provide written permission for their child to participate beforehand. All participants also had to consent to being audio recorded during the discussions.

### SD NRS

The SD NRS is a single-item, self-reported NRS scale designed to measure the degree of SD/sleep loss related to AD in patients with moderate-to-severe AD [18] for use in clinical research and potentially in clinical practice. The SD NRS asks patients to rate their sleep disturbances using the following question to the participant: On a scale of 0–10, with 0 being "no sleep loss related to the symptoms of atopic dermatitis" and 10 being "I cannot sleep at all due to the symptoms of atopic dermatitis", how would you rate your sleep last night? Given the day-to-day fluctuation in SD, the SD NRS was completed daily by the patients on an electronic device once daily in the morning throughout the clinical trial. Daily scores were averaged over a 7-day period from baseline to week 24 to derive an average weekly score. A minimum of four entries over a 7-day time frame was required to derive the average weekly score. If a patient had less than four diary entries in a week, the weekly average score was set to missing.

This measure was developed following the US FDA and International Society for pharmacoeconomics and Outcomes Research (ISPOR) guidance for developing PRO instruments [1, 19–21]. Its content validity has been demonstrated based on direct patient input in moderate-to-severe AD [18]. The psychometric properties of the SD NRS were also assessed using data from a phase IIb clinical trial in moderate-to-severe AD. These analyses found strong evidence for the reliability, validity, and responsiveness of the SD NRS for measuring day-to-day fluctuations of SD over time in patients with moderate-to-severe AD. This work has been published in a dermatological research journal [22].

### Study design

#### Phase IIb clinical trial

A multi-center, randomized, placebo-controlled, double-blind, parallel-group, dose-ranging study was carried out to evaluate the efficacy and safety of various doses of nemolizumab in 218 adults with moderate-to-severe AD and severe pruritus (NCT03100344) [23]. The primary efficacy endpoint of this study was the percent change in the Eczema Area and Severity Index (EASI) from baseline to week 24. Secondary efficacy endpoints include the SD NRS absolute and percent change from baseline to week 24. More details on the study design and other efficacy and safety endpoints are reported elsewhere [23].

#### Qualitative interview study

A hybrid concept elicitation/cognitive interviewing study to provide evidence of the content validity of the SD NRS in participants with moderate-to-severe AD, moderate-to-severe pruritus, and SD. The concept elicitation aimed at exploring patient experiences, with the objective of determining whether the SD NRS measured a concept of relevance and importance to patients with AD. Concept elicitation was followed by cognitive debriefing to assess whether the participants fully understood the SD NRS and to determine how easily they could complete the SD NRS. If sufficient time was available during the interviews, the patients were queried about what would constitute a meaningful change for them and the thresholds for a meaningful change on the SD NRS. Participants provided both numerical answer and qualitative descriptions on what constitute a meaningful change.

The qualitative study was conducted independently from the phase IIb study in the USA only and included 20 adult and 10 adolescent participants. Selection criteria similar to those of phase IIb study were used to recruit the qualitative sample to help support the equivalence of the two study samples (i.e., qualitative sample and phase IIb sample). Participants completed the SD NRS, and were interviewed during one-on-one, telephone sessions. Experienced and trained staff conducted interviews in US English using semi-structured interview guides.

### Estimation of MWIC thresholds based on the quantitative approach

Post-hoc analyses of Phase IIb clinical trial data were conducted using anchor-based and distribution-based approaches used to define MWIC threshold estimates for the SD NRS score. In the anchor-based approach, the two following analyses were conducted:

1. Longitudinal correlations between the SD NRS score and the selected anchor measures, i.e., the peak pruritus NRS (PP NRS), a single-item NRS designed to measure each subject’s worst itch intensity during the previous 24 h using a 11-point response scale (from 0 to 10), and the pruritus categorial scale (PCS), a single-item scale designed to measure each subject’s overall itch during the previous 24 h using a 4-point categorical response scale. Correlations between the SD NRS and each of these anchor measures were performed prior to performing the analyses to ensure that they were sufficiently large to proceed with the anchor-based analyses. A correlation threshold of 0.30 to 0.35 has been recommended as the minimum acceptable association between an anchor and a PRO change score [4].
2. Descriptive statistics of the observed change from baseline to week 16 in the SD NRS average weekly score were provided by anchor-based criteria. The following anchor criteria were applied: (a) a PCS score [24] at week 16 ≤ 1; and (b) change from baseline to week 16 in PP NRS score (≥ 4-point improvement) [24]. In addition, for each of these anchor measures, the distribution of the change from baseline to week 16 in the SD NRS score was plotted overall and by baseline SD severity subgroups (SD NRS score < 7 vs. SD NRS score ≥ 7).

The distribution-based methods were computed to support anchor-based methods. Distribution-based MWIC estimates included calculation of the standard error of measurement (SEM) and the half- and quarter-standard deviation (StD). The SEM was computed as the StD of an observed score related to its reliability (StD × square root [1 − intraclass correlation coefficient (ICC)]), where the ICC was from the SD NRS test–retest reliability in patients defined as stable based on the SCORAD sleep loss visual analogue scale (VAS). Primary analyses for anchor-based and distribution-based methods were conducted from baseline to week 16.

All analyses were conducted on all patients randomized in the phase IIb clinical trial who had SD NRS data at baseline. Analyses were performed using SAS version 9.4 (SAS Institute, Cary, NC, USA).

### Estimation of MWIC threshold based on the qualitative approach

Qualitative exploration of meaningful change threshold for the SD NRS was a secondary objective of the qualitative study. Participants’ perspectives on what change from the current day’s score they would consider to be the smallest improvement and that they would expect with a new treatment was elicited. Then, participants were further probed whether a 1-point, 2-point, or 3-point change would be a meaningful change to them and what that level of change meant to them. This approach provided the opportunity to distinguish between participants expectations related to the smallest change with a new treatment and the reality about a change that is meaningful to them.

All interviews were audio-recorded and transcribed by third-party professional transcription services. Quantitative sociodemographic and clinical data were collected to characterize the sample using descriptive statistics. Qualitative data collected in transcripts were analyzed using a deductive content analysis approach. The coding process was driven by the objectives of the study and consisted of tagging codes to segments of textual data to facilitate the comprehension of a large amount of data. A coding dictionary was developed to aid with the coding. Concept codes were used to capture the participants’ descriptions of their experiences with sleep problems and the impact on their everyday life. Specific codes related to meaningful changes were also used. Evidence of concept saturation was documented to ensure the adequacy of the sample size to address research questions.

All qualitative analyses were performed using ATLAS.ti, version 7.0 or higher.

The distribution of the desired change in SD NRS that participants would be satisfied with was plotted by age groups (adolescents < 18 years vs. adults >  = 18 years) and by SD severity subgroups on the day of the interview (SD NRS score < 7 vs. SD NRS score ≥ 7).

### Data triangulation to define a range of MWIC threshold estimates

The final phase of any mixed-methods design is the triangulation of the results and data interpretation. The triangulation consisted of comparing and integrating results from quantitative and qualitative approaches to define a range of MWIC threshold estimates (Fig. 1). Specifically, the findings from both the quantitative anchor-based and the distribution-based analyses were examined in light of qualitative findings from the smallest improvement and meaningful questions on the SD NRS to define a range of MWIC threshold estimates for SD NRS. In addition, distribution plots of the SD NRS score change (at week 16 for the quantitative approach and on the day of the interview for the qualitative approach) by SD severity subgroups were used to examine any variation on the MWIC according to the level of SD severity at the beginning of the study.

## Results

### Study samples

#### Phase IIb clinical trial

The analyses included 218 randomized patients who had an SD NRS score at baseline (Table 1). Mean age was 39.2 ± 15.2 years. Just over half of the patients (51.8%) were male, and most were White (75.8%) and not Hispanic or Latino (95.0%). Investigators assessed global severity as moderate (investigator global assessment [IGA] = 3) for 66% of patients and severe (IGA = 4) for 34%, and most patients (87.2%) reported having severe pruritus for ≥ 3 of the last 7 days based on the PCS. Baseline mean SD NRS was 7.8 ± 1.6 with only 19.7% of patients having an SD NRS score < 7.

**Table 1:** Participant Demographics and Clinical Characteristics at Baseline

Characteristic	Phase IIb data (QUAN data)	Concept elicitation interviews (qual data)	
	Adults (*N* = 218)	Adults (*N* = 20)	Adolescents (*N* = 10)
**Age (years), mean ± StD**	39.2 (15.2)	33.5 ± 12.8	14.1 ± 1.9
**Sex,** ***n*** **(%)**			
Male	113 (51.8)	8 (40)	5 (50)
Female	105 (48.2)	12 (60)	5 (50)
**Ethnicity,** ***n*** **(%)**			
Hispanic or Latino	11 (5.0)	5 (25)	2 (20)
Not Hispanic or Latino	207 (95.0)	15 (75)	8 (80)
**Racial background,** ***n*** **(%)**			
White	164 (75.2)	8 (40)	4 (40)
Black or African American	26 (11.9)	4 (20)	0 (0)
Asian	24 (11.0)	7 (35)	5 (50)
American Indian or Alaska Native	1 (0.5)	1 (5)	0 (0)
Other	3 (1.4)	0 (0)	1 (10)
**Pruritus**^**a**^, ***n*** **(%)**			
Mild	NR	0 (0)	0 (0)
Moderate	NR	7 (35)	3 (30)
Severe	NR	13 (65)	7 (70)
** ≥ 3 days with severe pruritus in the past 7 days, n (%)**	190 (87.2)	NR	NR
**EASI, mean ± StD**	25.6 ± 10.9	25.7 ± 11.5	26.3 ± 8.9
**IGA score, *n*** **(%)**^**b**^			
3 (moderate)	144 (66.1)	NR	NR
4 (severe)	74 (33.9)	NR	NR
**BSA score, mean ± StD**^**c**^	41.7 ± 18.6	NR	NR
**SCORAD, mean ± StD**	66.9 ± 11.6	72.5 ± 11.0	78.6 ± 15.6
**SD NRS score, mean ± StD**	7.8 ± 1.6	6.05 ± 2.15	5.77 ± 1.92
**SD NRS score,** ***n*** **(%)**			
< 7	43 (19.7)	10 (50.0))	5 (50.0)
≥ 7	175 (80.3)	9 (45.0%)	4 (40.0)
Missing	–	1 (5.0%)	1 (10.0)

^a^Based on the Pruritus Categorical Scale

^b^*N* = 215

^c^*N* = 217

*BSA* body surface area; *EASI* Eczema Area and Severity Index; *IGA* investigator global assessment; *NR* not reported; *SCORAD* Scoring Atopic Dermatitis; *SD NRS* sleep disturbance numeric rating scale; *StD* quarter standard deviation

#### Qualitative interview study

A total of 20 adult and 10 adolescent participants were enrolled across six clinical sites in the USA. The mean age of the adult participants was 33.5 years, and the majority were female (*n* = 12). The mean age of the adolescent participants was 14.1 years, and equal numbers were male and female (*n* = 5 each) (Table 1). Most participants were White or Asian, and most identified themselves as not Hispanic. According to the PCS, pruritus was severe for most participants (*n* = 13 adults, *n* = 7 adolescents); all patients had moderate-to-severe pruritus and sleep loss. Scores at screening for AD severity, pruritus, and SD NRS are reported in Table 1. Mean SD NRS score on the day of the interview was 6.05 ± 2.2 for adults and 5.78 ± 1.9 for adolescents, with an even proportion in each age group between those having an SD NRS score < 7 (*n* = 10 adults, *n* = 5 adolescents) and those having an SD NRS score ≥ 7 (*n* = 9 adults, *n* = 4 adolescents).

### Estimation of MWIC thresholds based on the quantitative approach

**Fig. 2:**
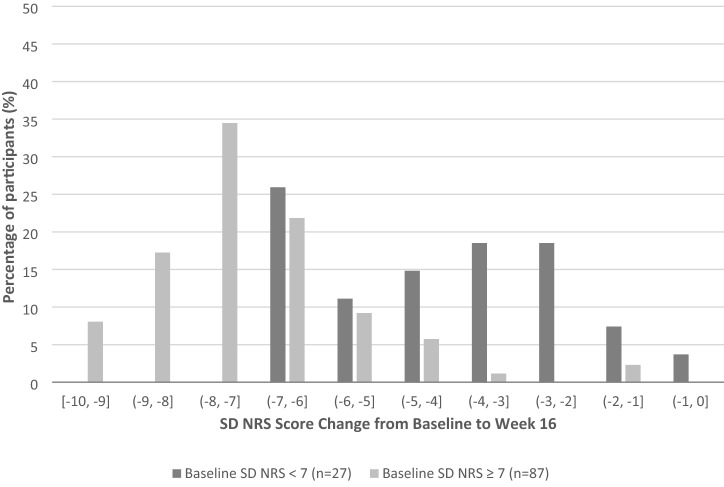
Phase IIb clinical trial data: SD NRS score change from baseline to week 16 on PCS responders by baseline SD NRS severity group (*N* = 114)**.** *PCS* pruritus categorical scale; *SD NRS* sleep disturbance numeric rating scale

Correlations between anchor measures and the SD NRS from baseline to week 16 using phase IIb data as well as the SD NRS score change from baseline to week 16 are presented in Table 2. Significant large correlations were seen between SD NRS score change from baseline to week 16, and PCS score change from baseline to week 16, and PP NRS score change from baseline to week 16 (*r* = 0.79 and *r* = 0.88, respectively). The PCS and PP NRS showed sensitivity to subjects’ changes in itch intensity with decreased SD NRS score (indicating an improvement in sleep disturbance) along with an improvement in itch (mean change score − 6.2 (StD = 1.99) and -5.6 (StD = 2.33), respectively). At week 16, the threshold for meaningful change was estimated to be 6.3 ± 2.1 points using PCS score as an anchor, and 6.7 ± 1.6 points using PP NRS score change as an anchor (Table 3). To establish minimum detectable changes in the SD NRS, distribution-based estimates were calculated using SD NRS data at week 16. These estimates resulted in an SEM of 1.58, an StD of 0.40, and half-standard deviation of 0.81. Additionally, among responder participants based on the PCS (i.e., PCS score ≤ 1 at week 16), their SD improved across all levels of SD improvement (Fig. 2). When looking at the distribution of the SD NRS score change by baseline SD severity group, participants with a baseline SD NRS ≥ 7 had higher decrease in SD NRS scores compared to the SD NRS < 7 group (Fig. 2). Furthermore, the most frequent change category in the SD NRS score was between a 6- to 7-point decrease for participants with a baseline SD NRS < 7 (25.9%) and between a 7- to 8-point decrease for participants with a baseline SD NRS ≥ 7 (34.5%) (Fig. 2).

**Table 2:** Phase IIb clinical trial data: correlations between anchor measures and SD NRS and mean change of the SD NRS from Baseline to week 16

Anchor		Correlation with SD NRS Score Change (Week 16—Baseline)^a^	*p*-value
PCS Score Change from Baseline to Week 16		0.79	< 0.001
PP NRS Score Change from Baseline to Week 16		0.88	< 0.001

	*N*	SD NRS Score Change (Mean StD) (Week 16—baseline)	*p*-value
**PCS Score Change from Baseline to Week 16**			< 0.0001
Improved	128	− 6.2 (1.99)	
Not Improved	44	− 1.7 (1.81)	
**PP NRS Score Change from Baseline to Week 16**			< 0.0001
Improved	152	− 5.6 (2.33)	
Not Improved	19	− 0.4 (1.21)	

^a^Spearman correlation coefficient

*NRS* numeric rating scale; *PCS* pruritus categorical scale; *PP* peak pruritus; *SD* sleep disturbance; *StD* standard deviation

**Table 3:** Phase IIb clinical trial data: MWIC estimates for the SD NRS score change between baseline and week 16

Definition type	Study	Definition	Meaningful or detectable change threshold estimate
Anchor-based	Phase IIb	≥ 4-point decrease in PCS score at Week 16, mean	6.3
		≥ 4-point decrease in PP NRS score at Week 16, mean	6.7
Distribution-based	Phase IIb	Quarter-standard deviation	0.40
		Half-standard deviation	0.81
		Standard error of measurement	1.58
Qualitative	Qualitative research	Smallest change considered satisfactory for > 80% of adults and adolescents (spontaneous report)	1–4
		Meaningful change endorsed for all adults and adolescents (when probed)	1–3

*MWIC* meaningful within-individual change; *NRS* numeric rating scale; *PCS* pruritus categorical scale; *PP* peak pruritus; *SD* sleep disturbance

**Fig. 3:**
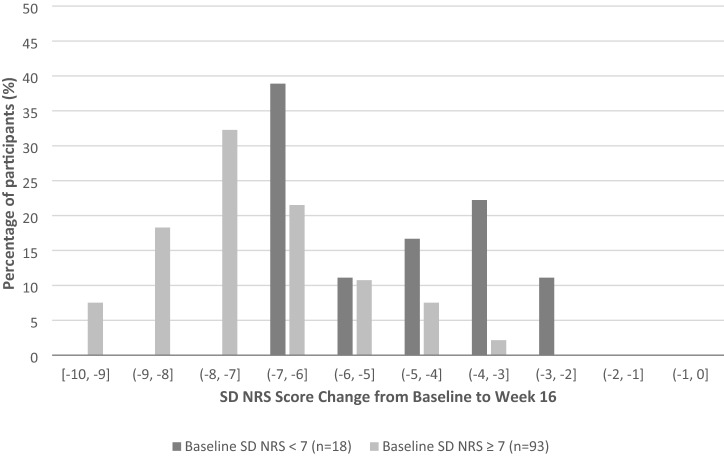
Phase IIb clinical trial data: SD NRS score change from baseline to week 16 on PP NRS responders by baseline SD NRS severity group (*N* = 111)**.** *NRS* numeric rating scale; *PP* peak pruritus; *SD* sleep disturbance

Similar results were observed between participants who were responders based on the PP NRS (i.e., PP NRS score change ≥ 4-point decrease at week 16) (Fig. 3).

### Estimation of MWIC threshold based on the qualitative approach

**Fig. 4:**
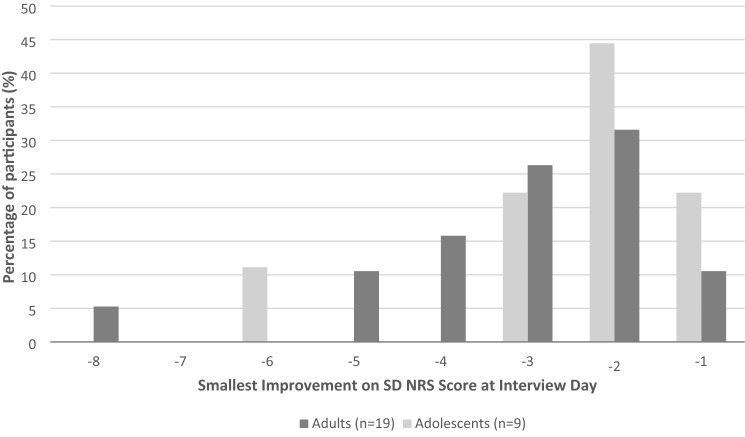
Interview study data: smallest improvement on SD NRS score at interview day in adults and adolescents**.** *SD NRS* sleep disturbance numeric rating scale

**Fig. 5:**
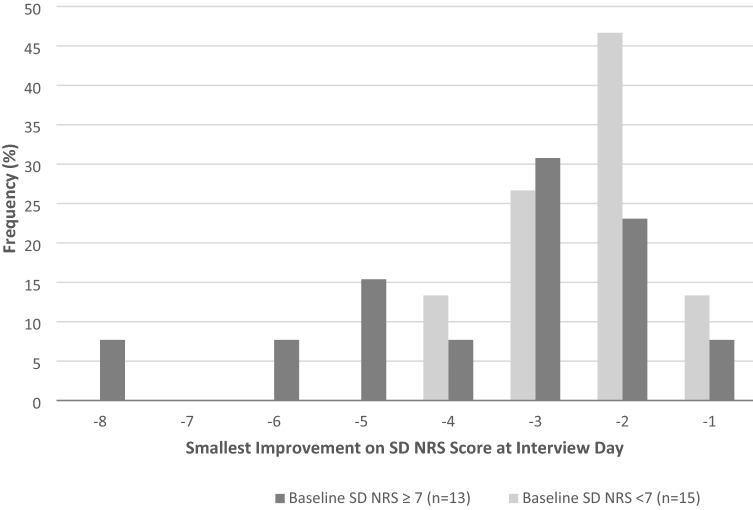
Qualitative interview study data: smallest improvement on SD NRS score at interview day by SD NRS severity group. *SD NRS* sleep disturbance numeric rating scale

Results from qualitative exploration of MWIC threshold are presented in Table 4. A total of 28 participants (19 adults and 9 adolescents) out of 30 were asked about the smallest improvement they would be satisfied or content with on the SD NRS. Of these, most (69% of adults and 88% of adolescents) indicated a change of one to three points, with a two-point change being the most frequent response in both groups (32% of adults and 44% of adolescents) (Fig. 4). A greater proportion of adult participants (32%) indicated that they would expect higher than a three-point change on the SD NRS to be satisfied or content, compared to 11% (one participant) in the adolescent subgroup. Additionally, a greater proportion of adult and adolescent participants in the SD NRS ≥ 7 group (38%) indicated that they would expect a change higher than a three-point improvement on the SD NRS to be satisfied or content, compared to 13% in the SD NRS < 7 group (Fig. 5). Additionally, a total of 28 participants (17 adults and 10 adolescents) out of 30 were probed on meaningfulness of 1-, 2-, 3-point change. All respondents endorsed a change of one to three points to be meaningful, with a one-point change being the most endorsed response in both groups (76% of adults and 80% of adolescents), suggesting that a meaningful change would not necessarily be a change that the patients would be satisfied or content with a new treatment. When probed about what each level of change means to them, some participants described a one-point improvement as *“sleep a little better”* or be able to *“sleep more hours without dealing with a medication.”* For some participants, they described a two-point improvement as *“tremendous”* and further explained that *“if you were losing one hour of sleep every night, you would probably be losing 30 min of less”* and another mentioned *“I don’t have to wake up in the middle of the night.”* Finally, a 3-point improvement was described by some participants as *“not having to take a shower in the middle of the night”* or *“change from waking up a couple of times to being awake for the duration of the night.”*

**Table 4:** Meaningful change in SD NRS

Assessment	Adults	Adolescents	Illustrative quotes	
			Adults	Adolescents
**Smallest improvement that participants would be satisfied or content with**	*N* = 19	*N* = 9	*“…any improvement is good…”*	*“I just want to be able to stay asleep once I fall asleep. I don’t want to have to wake up and deal with it all over again once I already fell asleep”*
1 point	11%	22%		
points	32%	44%		
3 points	26%	22%		
4 points	16%	–		
5 points	11%	–		
6 points	–	11%		
8 points	5%	–		
**Level of improvement that would be meaningful**	*N* = 17	*N* = 10	*“[An improvement of 1 point]: Maybe I might sleep a little bit better”* *“[An improvement of 1 point]: Maybe I have sleep for more hours without dealing with a medication”* *“[An improvement of 2 points] means I could go to sleep better and that means I don’t have to wake up in the middle of the night knowing that I’m scratching myself or that means I can put makeup on and it will cover my face”* *“[An improvement of 3 points] 6 to 3 would probably be way, way easier. You probably wouldn’t have to worry about showering like day and night”*	*“I just feel like the 1-point difference is kind of understandable for just a weekly basis”* *“[An improvement of 3 points] I think I would definitely recognize that, because, I mean, like it's just a big change from waking up a couple times to being awake for the duration of the night”*
1 point	76%	80%		
2 points	18%	10%		
3 points	6%	10%		

*SD NRS* sleep disturbance numeric rating scale © Galderma

### Data triangulation to define a range of MWIC threshold estimates

**Fig. 6:**
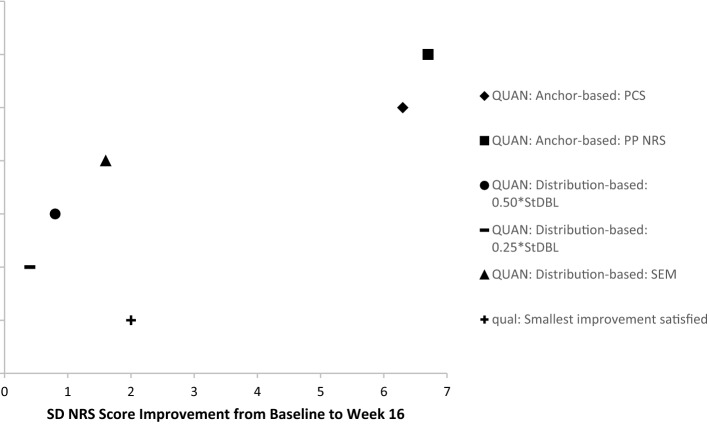
Results of MWIC threshold estimates based on quantitative anchor- and distribution-based analyses and qualitative analyses. *MWIC* meaningful within-individual change; *PCS* pruritus categorical scale; *NRS* numeric rating scale; *PP* peak pruritus; *SD* sleep disturbance; *SEM* standard error of mean

Results of the anchor-based, distribution-based, and qualitative-based analyses were triangulated to obtain a preliminary MWIC range of thresholds (Fig. 6). Results from the quantitative approach suggested that a 2- to 6-point decrease in the SD NRS is a meaningful improvement, while results from qualitative findings revealed that most patients (69% of adults and 88% of adolescents) expect a change of one- or three-point change with a new treatment. However, the majority (90% in each population) of them endorsed when probed a one- or two-point change being meaningful. Qualitative findings also suggested that the more severe the sleep disturbance the day of the interview, the higher the expectations for change on SD NRS.

## Discussion

The objective of this paper was to present the MMR design applied to establish MWIC estimates of the 11-point SD NRS in adults and adolescents with moderate-to-severe AD in the context of a drug development program. An explanatory sequential design was applied in which data from a phase IIb trial was primarily used to define a range of MWIC threshold estimates supported by data from a qualitative study. Although it’s acknowledged that the qualitative study population and phase IIb study population are separate individuals, both involved individuals with moderate-to-severe AD. Findings from the quantitative anchor-based and distribution-based methods using data from the phase IIb trial suggested a range of 2–6-point reduction in the SD NRS as a meaningful improvement for adults with moderate-to-severe AD. The findings from the qualitative study suggested a 2-point and 3-point reduction in the SD NRS as a meaningful improvement for adolescents and adults, respectively. However, qualitative results revealed that while most adult and adolescents (> 90%) endorsed a lower change (one- or two-point change) being meaningful when specifically probed, a proportion of participants still had higher expectations for change (> 3-point) with a new treatment, particularly those with a SD NRS ≥ 7 score the day of the interview, suggesting that, although meaningful, a change of 2-point and 3-point would not necessarily be a change that the patients would be satisfied or content with.

The triangulation of results confirmed that a range of MWIC threshold estimates of 2- to 6-point reduction in the SD NRS was a meaningful improvement for the target population of adults and adolescents with moderate-to-severe AD. The upper range of the proposed MWIC estimate (6-point change) is above the MWIC threshold of a 3- to 4-point change generally accepted for a single-item 11-point NRS scale [24, 25]. However, the 2- to 6-point range is supported by the fact that the phase IIb trial included an adult population with severe SD at baseline (mean baseline SD NRS score of 7.8 with about 80% of the participants having a baseline SD NRS score ≥ 7) allowing for a larger improvement at the end of the treatment period. On the other hand, the lower range of the proposed MWIC estimate (2-point reduction) was mainly driven by the qualitative study population, including adults and adolescents who reported less severe SD the day of the interview (mean SD NRS score of 5.96 with about 54% of the participants having a baseline SD NRS score < 7). In addition, the MWIC was estimated in the phase IIb trial from data collected daily over a 24-week period based on each participants’ actual experience of SD, while in the qualitative study the MWIC was estimated based on each participants’ desired change in SD NRS based on the interview day score. Differences in the severity of SD between the “QUAN” and “qual” populations, the age groups (phase IIb only in adults), the research context (clinical trial vs. observational, non-drug study), as well as on the data collection approach for MWIC estimates (actual vs. desired SD NRS change) may explain the differences in quantitative and qualitative results and the large range of estimates for MWIC. However, some of these limits also strengthen this approach as the results revealed that MWIC estimates may vary across age groups (adults vs. adolescents), but also across SD severity groups at baseline and, therefore, the MWIC estimates should be further scrutinized by age and SD severity groups using another MMR design involving additional quantitative and qualitative data.

The first stage involving this explanatory sequential MMR design was successfully applied to define a preliminary range of MWIC threshold estimates on the self-reported SD NRS in the target population. As a next step, a second MMR stage design will be used to narrow the MWIC threshold range, possibly to a single threshold, using both quantitative and qualitative data from the same study sample in both adult and adolescent AD populations.

The use of multistage MMR design which consists in using several mixed-methods projects conducted either concurrently or sequentially with the aim of addressing a single research question [26] offers the possibility of using pluralistic approaches to collect and analyze data following a pragmatist tradition [27; 28].

The iterative data collection approach, as well as the triangulation of multiple methodologies and data sources, will improve the overall robustness of the design for establishing an MWIC threshold and classifying an individual as a responder [29].While establishing an MWIC threshold through anchor-based methods supplemented by exit interview data collected in the context of a clinical trial, or a standalone qualitative study, has already been used and published [5; 6; 30], to our knowledge the use of a multistage MMR design to estimate a final MWIC threshold or a final range of MWIC thresholds remains a novel approach.

This multistage MMR design, integrating multiple qualitative and quantitative methods and allowing for integration and interpretation of data at each stage, is best suited when various types of data sources (i.e., data from clinical trials and external qualitative studies) are available. This emerging approach leverages standardized, generalizable data such as clinical trial data (i.e., phase IIb and phase III clinical trial data) combined with the richness and subjective insights collected from patients (i.e., concept elicitation and clinical trial exit interviews). Additionally, it strengthens the MWIC threshold findings, better contextualizes and explains the results, and minimizes the weaknesses of a single method or MMR single stage.

This study has several limitations. As published in Puelles et al., 2021 [22], the impact of the SD NRS assessing the less severe spectrum of SD was not taken into account in this analysis. However, the qualitative findings [18] reported that the SD NRS is anticipated to perform satisfactory among participants with less severe SD. Additionally, the study inclusion and exclusion criteria and demographic profile of patients who agreed to participate in this clinical trial may impact the generalizability to a broader population or other disease indication. Both the qualitative study data and clinical trial data for the SD NRS and anchors used in this analysis are based on self-report, creating a small, but minimal, risk to accuracy based on human error. In addition, we have used the mean change as a threshold estimate for MWIC despite several known issues and criticisms [31; 32]. We believe that our mean-change approach is appropriate and of standard practice; however, we acknowledge that there was an implicit assumption that all improvements were the same across individuals within a group, which is not necessarily the case in reality.

## Data Availability

The datasets generated during and/or analysed during the current study are not publicly available due to data sharing policies but are available from the corresponding author on reasonable request.
